# The natural product 2,4,6-tribromoanisole is the predominant polyhalogenated compound in representative Australian passive air samples

**DOI:** 10.1007/s10661-025-14638-7

**Published:** 2025-10-24

**Authors:** Sina Schweizer, Xianyu Wang, Chris Paxman, Jochen F. Mueller, Walter Vetter

**Affiliations:** 1 Institute of Food Chemistry, Department of Food Chemistry (170B), University of Hohenheim, Stuttgart, 70599 Germany; 2 University of Queensland, Queensland Alliance for Environmental Health Sciences, Brisbane, 4102 Australia

**Keywords:** Halogenated natural product, Persistent organic pollutant, 2,4,6-Tribromoanisole, Passive air sample, Australia

## Abstract

Passive air samplers are well-suited for monitoring persistent organic pollutants (POPs) in ambient air. While the presence of POPs had been documented in Australian air, no data existed on structurally similar, halogenated natural products (HNPs), although these were frequently found in marine biota samples from Australia at levels exceeding those of anthropogenic POPs. This study reports quantitative data of the HNP 2,4,6-tribromoanisole (2,4,6-TBA) along with three POPs (polychlorinated biphenyls (PCBs) 153 and 138 as well as hexachlorobenzene (HCB)) in six selected passive air samples from different Australian regions (islands, coastal cities, and inland). For the most abundant HNP, 2,4,6-TBA, time-averaged concentrations for one year were determined at up to 420 pg/m^3^ (One Tree Island), indicating its predominant natural production in the Great Barrier Reef (GBR). High concentrations of 2,4,6-TBA (17 pg/m^3^), even in the remote inland sample (~ 800 km from the sea), led to the conclusion that the marine-derived 2,4,6-TBA was transported over long distances in air and can be found ubiquitously in Australian air. Even in the coastal cities of Brisbane and Darwin, 2,4,6-TBA levels were comparable to those of the PCBs. The HNP 2,3,3',4,4',5,5'-heptachloro-1'-methyl-1,2'-bipyrrole (Q1) was also detected in air from two islands. Its presence in air from One Tree Island was in line with expectations, given the high levels in marine mammal samples from the GBR. In direct comparison, the ~15 times higher ratio of Q1/2,4,6-TBA in air from Phillip Island indicated Q1 could be even more abundant in this marine region than in the GBR.

**Supplementary Information:**

The online version contains supplementary material available at 10.1007/s10661-025-14638-7.

## Introduction

Volatile and semi-volatile persistent organic pollutants (POPs) and compounds of similar properties tend to partition into the air, in which they can be transported to remote areas and thereby get distributed in the environment (Fuhrimann et al., 2020; Wang et al., 2015). Monitoring ambient air samples can help to identify (i) pollution trends (long-term measurements), (ii) the pollution status of a given region, and (iii) the entry of new POPs and POP-like contaminants (comparative measurements) into the environment (Wang et al., 2015; Seethapathy et al., 2008). One well-suited method for monitoring the levels of persistent organic pollutants (POPs) in the atmosphere is using passive air samplers (Wang et al., 2015). Passive air samplers take advantage of a sorbent on which target compounds get enriched during deployment (Bartkow et al., 2005; Fuhrimann et al., 2020). For passive air samplers, adsorbents are used that serve as zero sinks, which means that the concentration of the analytes on the sorbent surface is close to zero (Seethapathy et al., 2008). In this scenario, the quantity being transferred from the air into the sampler over a given period relative to the mean concentration in the air over that time is proportional to a constant sampling rate, and this enables the determination of time-weighted average concentrations (Seethapathy et al., 2008). So, if the compound-specific sampling rate has been determined, the POP levels quantified on the adsorbent can be transferred into concentrations in the atmosphere (Górecki & Namieśnik, 2002). In addition, active sampler types, which actively pump the air through the sorbent, can also be used to determine the level of analytes in ambient air (Fuhrimann et al., 2020; Li et al., 2023). In either case, such monitoring programs of passively or actively sampled ambient air samples can document the extent of the release of the analytes under study from different sources by comparing samples from different sites (Wang et al., 2015).

**Fig. 1:**
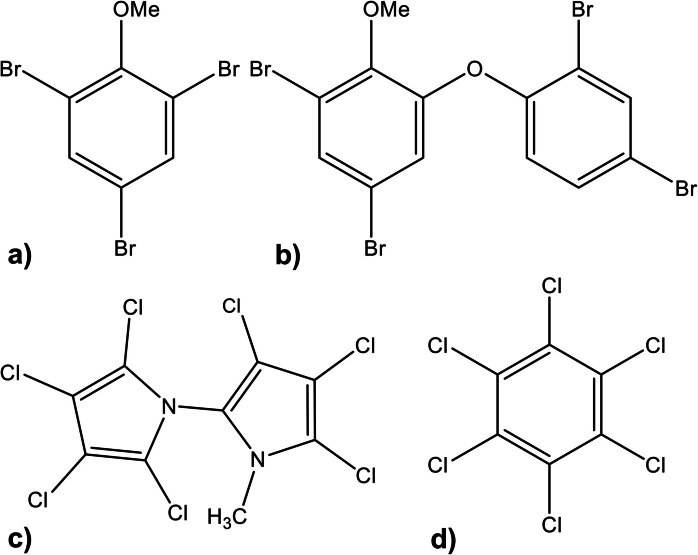
Chemical structures of the halogenated natural products **a** 2,4,6-tribromoanisole, **b** 2′-methoxy-2,3′,4,5′-tetrabromodiphenyl ether (2′-MeO-BDE 68 or BC-2), and **c** 2,3,3´,4,4´,5,5´-heptachloro-1'-methyl-1,2'-bipyrrole (Q1), as well as **d** the persistent organic pollutant hexachlorobenzene (HCB)

POP levels in Australian air were already investigated using passive air samplers equipped with polystyrene-divinylbenzene resin (XAD) adsorbent (Wang et al., 2015). XAD is characterized by a high uptake capacity of semi-volatile organic compounds before reaching equilibrium after a very long time (Shunthirasingham et al., 2010). This allows the passive air samplers to be deployed for a year and then to determine the annually averaged POP levels (Shunthirasingham et al., 2010). So far, halogenated natural products (HNPs) had not been analyzed in Australian ambient air samples. Produced by marine algae, sponges, and bacteria, some HNPs are structurally similar to POPs and therefore have similar properties such as persistence, bioaccumulation, and toxicity (Fig. 1, Tab. S1) (Gribble, 2023, 2024).

Although the existing data are disjointed, several HNPs were found to be particularly abundant in Australian marine areas, as repeatedly verified by the analysis of matrices ranging from marine mammals (Vetter et al., 2001) to passive water samplers (Gaul et al., 2011; Vetter et al., 2018). Some HNPs have also already been detected in air from other sites, partly with different trends than POPs (Bidleman et al., 2017a, 2017b, 2019, 2020; Kim et al., 2014; Melcher et al., 2008; Paul & Pohnert, 2011; Pfeifer et al., 2001; Wittlinger & Ballschmiter, 1990). Therefore, the Arctic Monitoring and Assessment Program (AMAP) classified some HNPs as chemicals of emerging Arctic concern in 2016 (Arctic Monitoring and Assessment Programme).

The present study aimed to verify the presence of HNPs in Australian ambient air samples. Due to their known presence in Great Barrier Reef (GBR) samples, we selected three passive air samplers deployed on Australian islands and compared them with those from two coastal cities and one remote inland site. Since many environmentally relevant HNPs are (partly) brominated, we first applied gas chromatography with electron-capture negative ion mass spectrometry operated in selected ion monitoring mode (GC/ECNI-MS-SIM) with a focus on low mass fragment ions, which enable the detection of virtually all polybrominated and polychlorinated compounds (Vetter, 2001). For the HNPs detected this way, the compound-specific sampling rate in the standard air XAD sampler had recently been experimentally estimated for 2,4,6-TBA (Li et al., 2023), so the mass accumulated in the samplers and deployment period were used to estimate a time-averaged concentration of 2,4,6-TBA in the air at those sites. To better classify the results, we also quantified selected anthropogenic POPs, namely hexachlorobenzene (HCB) and the polychlorinated biphenyls (PCBs) 2,2’,3,4,4’,5’-hexachlorobiphenyl (PCB 138) and 2,2’,4,4’,5,5’-hexachlorobiphenyl (PCB 153), whose sampling rates are relatively well characterized (Armitage et al., 2013; Wania et al., 2003).

## Material and methods

### Chemicals and standards

Chemicals and standards used for the identification and quantification of HNPs in the air sampler samples were described in detail previously (Schweizer et al., 2024). In brief, *i*-octane, *n*-hexane (for pesticide residue analysis), and ethyl acetate (distilled before use) were obtained from Honeywell Riedel-de Haën (Seelze, Germany), Chemsolute Th. Geyer (Renningen, Germany), and Sigma-Aldrich (Steinheim, Germany). Silica gel G60 (for column chromatography grade) and sodium sulphate (> 99%, water free, p.a.) were purchased from Carl Roth (Karlsruhe, Germany). Reference standards and internal standards were isolated, synthesized, or obtained from Sigma-Aldrich (Steinheim, Germany), Dr. Ehrenstorfer (Augsburg, Germany), and AccuStandard (New Haven, CT, USA). Specifically, 2,4,6-TBA was purchased from Sigma-Aldrich (Steinheim, Germany), while the PCB indicator mix and HCB were from Dr. Ehrenstorfer (Augsburg, Germany). The other quantified HNPs, 2′-methoxy-2,3′,4,5′-tetraBDE (2′-MeO-BDE 68 or BC-2) and 2,3,3',4,4',5,5'-heptachloro-1'-methyl-1,2'-bipyrrole (Q1), were synthesized in our working group (Vetter et al., 2003; Wu et al., 2002).

### Sampler characteristics and deployment plan

**Fig. 2:**
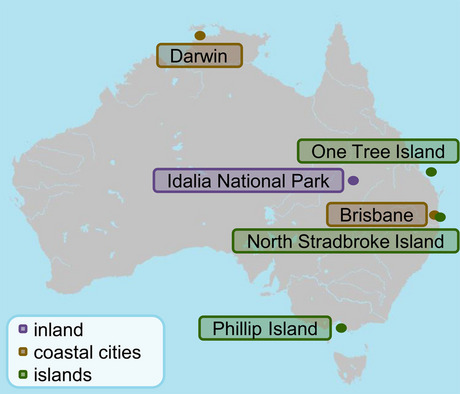
Map of Australia (1:45,000,000) showing the different sampling sites of the air samples

The six sites were sampled as part of a monitoring program on temporal and spatial trends of organic pollutants in Australia by the Queensland Alliance for Environmental Health Sciences (Brisbane, Australia). Before deployment, the passive air sampler materials and 10 cm long empty tubes were first heated to 450 °C overnight, followed by rinsing with methanol, *n*-hexane, and acetone. The tubes were then filled with approximately 10 g of polystyrene-divinylbenzene resin (XAD) type 2 (size: 20–60 mesh, surface: ~ 300 m^2^/g), with glass wool added at the top and bottom. Prepared 10 cm long XAD mesh tubes (area of 62.5 cm^2^ (Wang et al., 2015)) were hung up in the housing and deployed at the six sampling sites at 1.5 m deployment height for ~ one year (Fig. S1). Sites included islands, coastal cities, and rural locations (Tab. S2). For the coastal sites, there was the possibility for sea spray in the air. As Wania et al. have shown through wind tunnel experiments, water in the air is able to reach the adsorbent in the passive sampler, but there is no direct air flow through the shelter, leading to a constant uptake rate for different wind speeds (Wania et al., 2003). Specifically, two samples were from urban coastal cities (Brisbane (BRI) and Darwin (DAR)), three from sparsely populated islands (North Stradbroke Island (NSI), distance to Brisbane/coast, ~ 30 km/~ 10 km, One Tree Island (OTI) from the southeast of the GBR, distance to coast, ~ 90 km, and Phillip Island (PHI) close to Melbourne, distance to Melbourne/coast, ~ 70 km/~ 10 km), as well as one from the Australian inland with no settlement nearby as a control sample (Idalia National Park (IDA), distance to the sea, ~ 800 km) (Fig. 2). Additionally, a laboratory blank sample (BLK; a clean sealed XAD tube was stored at room temperature throughout the sampling year and at −20 °C afterwards) was analyzed as well. All samples besides DAR (sampled in 2011) were from 2020.

### Sample extraction and clean-up

After the deployment, aliquots (~ 4 g, exactly weighed) of the homogenized XAD were taken in duplicate from the passive samplers and spiked with 20 ng of the recovery standard perdeuterated *α*-hexachlorocyclohexane (*α*-PDHCH). Lipophilic compounds were gained by accelerated solvent extraction (ASE 350, Dionex/Thermo Fisher Scientific, Waltham, MA, USA) with (i) 26.4 mL cyclohexane/ethyl acetate (46/54, *w/w*) and (ii) 26.4 mL ethyl acetate using the parameters of Wu et al. (Wu et al., 2020). Extracts (i) and (ii) were combined, the solvent was carefully changed to *n*-hexane in a calibrated pointed flask, and the volume was adjusted to ~ 1 mL using a rotary evaporator. The concentrated sample extracts were purified by adsorption chromatography using 3 g silica gel deactivated with 30% water and elution with 60 mL* n*-hexane (Wu et al., 2020). After adjusting the volume to exactly 1 mL, 500 µL were separated and concentrated tenfold (50 µL) with 20 ng of the internal standard 6′-MeO-BDE 66 (BCIS) being added as an instrumental standard before adjusting to 50 µL. HNPs and POPs were quantified by GC/ECNI-MS-SIM.

### Instrumentation

Quantification by GC/ECNI-MS-SIM was conducted with the Agilent 7890/5975C (Waldbronn, Germany) instrument and operation parameters previously described in detail (Schweizer et al., 2025). In brief, the ion source and quadrupole temperatures were set to 150 °C and the transfer line temperature to 300 °C. The applied capillary column (30 m × 0.25 mm i.d. × 0.25 μm d_f_ Optima 5 MS; Macherey-Nagel, Düren, Germany) was temperature-programmed from 50 °C (held 1 min) at 10 °C/min to 300 °C (held for 14 min).

### Calculation of HNP and POP concentrations in the air

Concentrations of the different analytes in the air from the amount in the sampler could be determined for 2,4,6-TBA, HCB, PCB 138, and PCB 153, whose sampling rates were known. Therefore, the total content in the sampler [pg] (Tab. S2) was divided by the length of deployment [days] multiplied by the sampling rates for 2,4,6-TBA (0.42 m^3^/day (Li et al., 2023)) (Tab. S3), HCB (0.40 m^3^/day (Li et al., 2023)), PCB 138 (0.55 m^3^/day (Armitage et al., 2013)), and PCB 153 (0.55 m^3^/day (Armitage et al., 2013)) (Eq. 1).

1 $$Concentration\;in\;the\;air\;\lbrack pg/m^3\rbrack\;=\frac{Content\;in\;the\;sampler\;\lbrack pg\rbrack}{(Length\;of\;deployment\;\lbrack days\rbrack\;\cdot\;Sampling\;rate\;\lbrack m^3/day\rbrack)}$$

### Quality assurance

The glassware used for the analysis was cleaned with detergent, demineralized water, acetone, and cyclohexane/ethyl acetate (46:54, *w*/*w*). In addition to the laboratory blank value of the XAD tube, two reagent blanks were prepared in the same way as the samples, but without a sample added. The recovery rate of *α*-PDHCH ranged from 88 to 121% for the analyses carried out and thus within the range (70–130%) considered acceptable. Contents of the analytes were determined using the respective reference standards and not corrected by the recovery rate. The contents determined for the duplicate determinations differed generally by less than 20%. Therefore, the mean values were calculated, and only these will be discussed in the following. The limits of detection (LOD, signal-to-noise ratio of three) of the examined HNPs were between 0.07 and 6 pg, and those of the POPs were 0.01–10 pg (Tab. S4). None of the quantified analytes was present in the blank samples.

## Results and discussion

### Air concentrations of the halogenated natural product 2,4,6-TBA

**Fig. 3:**
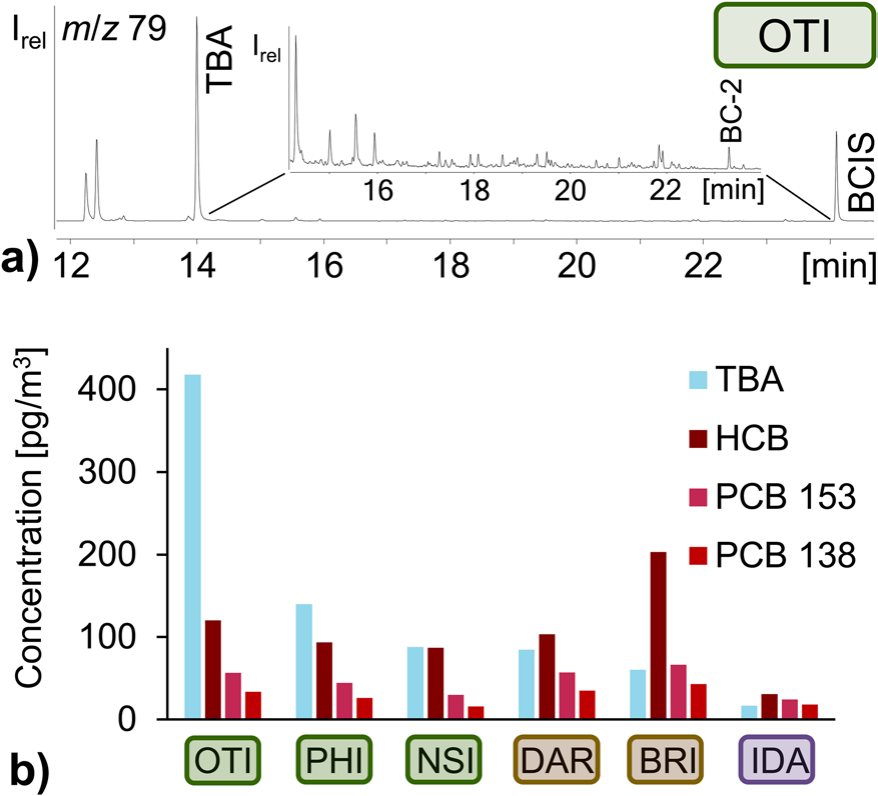
Extracted ion chromatogram **a** of *m*/*z* 79 from the GC-ECNI-MS-SIM measurement of the air sample from One Tree Island as well as **b** air concentrations [pg/m^3^] of the HNP TBA (blue) and POPs HCB, PCB 153 and PCB 138 (red colors) determined in the air samples from different regions (inland (purple box), coastal cities (brown box) and islands (green box)) of Australia. With: OTI: One Tree Island, PHI: Phillip Island, NSI: North Stradbroke Island, DAR: Darwin, BRI: Brisbane, and IDA: Idalia National Park

GC/ECNI-MS-SIM chromatograms (low mass fragment ions (Vetter, 2001)) unequivocally indicated several polybrominated compounds, with 2,4,6-TBA representing the most prominent peak (Fig. 3a). This was in agreement with air samples collected in other areas (Bidleman et al., 2017a; Melcher et al., 2008). Due to the known sampling rate (0.42 m^3^/day (Li et al., 2023)), 2,4,6-TBA quantities in the samplers (2.3–62 ng, Tab. S2) could be used to estimate an average atmospheric concentration for the period of sampler deployment (Eq. 1). The resulting highest air concentration of 2,4,6-TBA of 420 pg/m^3^ (average value for the whole year) was determined at One Tree Island (OTI, GBR) (Fig. 3, Tab. S3).

**Fig. 4:**
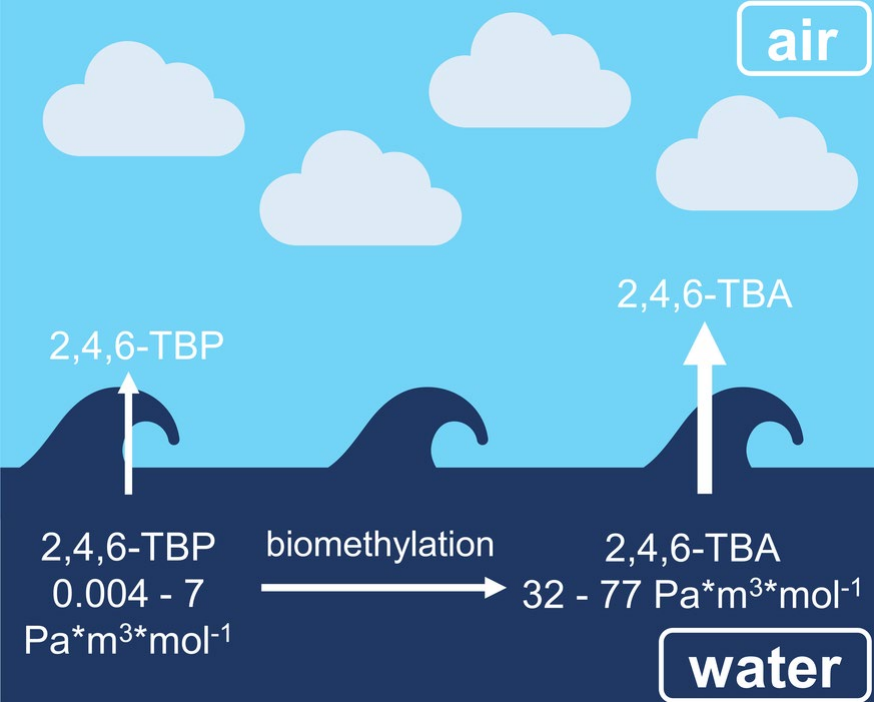
Illustration of the formation of 2,4,6-TBA from 2,4,6-TBP in the marine environment, as well as the partitioning of them into the air with the Henry’s law constants (Sander, 2023) indicated

2,4,6-TBA is unique in that its natural producer(s) are not known until today (Atlas et al., 1986; Vetter, 2006). However, there is a consensus that it is formed from 2,4,6-tribromophenol (2,4,6-TBP) by *O*-biomethylation (Atlas et al., 1986; Neilson et al., 1983; Vetter, 2006). While 2,4,6-tribromophenol (2,4,6-TBP) is a prominent HNP in marine biota (Chung et al., 2003; Whitfield et al., 1999), anthropogenic sources are known as well (Atlas et al., 1986; Löfstrand et al., 2010; Vetter, 2006). Different temporal trends throughout the year in coastal air samples from Norway indicated that POPs and bromoanisoles originated from different sources and that 2,4,6-TBA was mainly produced naturally (Melcher et al., 2008). In fact, 2,4,6-TBP is usually one of the most prominent organobromine compounds in marine water (Bidleman et al., 2019; Michałowicz et al., 2022), but as their Henry’s law constants (2,4,6-TBP: 0.004 to 7 Pa*m^3^*mol^−1^, 2,4,6-TBA: 32 to 77 Pa*m^3^*mol^−1^ (Sander, 2023)) show, it partitions considerably less into the atmosphere than 2,4,6-TBA, which is less polar and more volatile (Fig. 4).

The 25 times higher 2,4,6-TBA amount in air from One Tree Island (OTI) compared to the remote inland sample (Idalia National Park, IDA; 17 pg/m^3^, Fig. 3b), which was sampled in the same year (2020), produced strong evidence for its natural production in the GBR. Even more since 2,4,6-TBA levels in air from other marine areas were only in the low pg/m^3^ range, e.g., in Norway (0.1–37 pg/m^3^, active samplers) (Bohlin-Nizzetto et al., 2020; Melcher et al., 2008), the Baltic Sea (18–44 pg/m^3^, passive samplers) (Bidleman et al., 2017b), the Canadian Arctic Archipelago (6–39 pg/m^3^, active samplers) (Wong et al., 2011) with an increase of concentrations over time (Hsu et al., 2025), and even the Antarctic (0.1 pg/m^3^, passive sampler at Casey Station; 7.1 pg/m^3^, active sampler at Troll Atmospheric Observatory) (Bengtson Nash et al., 2021). The concentrations determined by active samplers usually represent a much shorter time period than those common for passive samplers and therefore cannot be directly compared with those of passive samplers (although their order of magnitude should nevertheless be in the same range). The fast sampling with active samplers enables the setup of measurements with higher temporal resolution, e.g., by taking one active air sample a week over a year or longer, giving the annual distribution of a given POP or HNP (Bohlin-Nizzetto et al., 2020). Data from such a campaign can also be used to calculate the annually averaged air concentrations (Bohlin-Nizzetto et al., 2020). For example, an annual average 2,4,6-TBA content of 8.2 pg/m^3^ was determined at Zeppelin station (Ny-Ålesund, Arctic) in 2020, while the weekly averaged concentrations ranged between 0.99–23 pg/m^3^ (Bohlin-Nizzetto et al., 2020). Clearly, the weeks and months with the highest POP and HNP load have an over-proportional impact on the annual average value. Multiple studies on air samples from Norway showed that the 2,4,6-TBA levels varied throughout the year, with the highest levels in summer and autumn, which was assumed to be due to the algal bloom during this time, along with higher temperatures (Bohlin-Nizzetto et al., 2020; Melcher et al., 2008). Additionally, spatial trends of 2,4,6-TBA, with lower levels in the Northern Hemisphere than in the Southern Hemisphere and a weak correlation with the air temperature, have already been reported in the literature (Atlas et al., 1986; Arctic Monitoring and Assessment Programme; Bengtson Nash et al., 2021; Melcher et al., 2008). Moreover, it has been shown for Grouse Mountain that with increasing altitude, 2,4,6-TBA concentration decreased in the air and increased in soil (Zhang et al., 2025). Therefore, the lower concentrations in reported data were at least partly due to the much higher air temperature at the Australian sites. For instance, the average annual temperature (2020) on One Tree Island was 24.6 °C (Anonymous, 2025b) and 18.8 °C on Phillips Island in the southernmost part of the Australian mainland (Anonymous, 2025c). In comparison, the mean temperature (2020) was 5.2 °C in the north of Norway (Anonymous, 2025a) and 7.0 °C in Finland (Anonymous, 2025d), while in Antarctica (King George Island), it was −2.8 °C (1947–1995) (Ferron et al., 2004). In line with that, higher 2,4,6-TBA levels were determined in the tropic eastern North Atlantic (170 pg/m^3^) (Führer et al., 1996), which was in the range of the sample with the second highest content, i.e., Phillip Island (PHI) with 140 pg/m^3^ 2,4,6-TBA (Tab. S3, Fig. 3b). Considering the distance to sea (800 km from the coast), the low(er) but still comparably high 2,4,6-TBA concentration of 17 pg/m^3^ in the inland sample (Idalia National Park, IDA) becomes even more striking (see below).

The 2,4,6-TBA content (2020) at One Tree Island (OTI) in the GBR exceeded all the previous reports in air samples on this compound. In passive water samples from the GBR, 2,4,6-TBA was detected at 6–3,300 pg/L (mean 170 pg/L), consistent with the high contents in the air (Vetter et al., 2018). Of the studied islands, One Tree Island (OTI, GBR) had the longest distance from the continental coast (~ 90 km) (Fig. 2). Our observation that the highest levels of HNPs were observed at an island and the lowest levels in the remote inland sample (Fig. 3b) agreed with the fact that HNPs are produced by marine organisms like sponges, algae, and bacteria (Bidleman et al., 2020). North Stradbroke Island (NSI) featured only 88 pg/m^3^, which was only slightly higher than the content of 60 pg/m^3^ in Brisbane (BRI). However, both sites are just ~ 30 km from each other, and Brisbane is also located on the coast (Fig. 2).

Our data confirmed high 2,4,6-TBA levels in coastal marine areas, most notably in the GBR (Pfeifer et al., 2001). Differences in the 2,4,6-TBA levels in air samples from an island compared to the inland were already found in a study of the Bothnian Bay region (Bidleman et al., 2017b). Additionally, bromoanisole levels were generally higher in coastal areas than in the inland, which underlines their natural (marine) origin (Bidleman et al., 2020; Bidleman et al., 2023; Zhan et). Clausius-Clapeyron (CC) plots (logarithm of the partial vapor pressure vs. reciprocal temperature), which are used to determine the importance of local surface–air exchange, have also shown in a number of locations that in coastal areas local exchange was responsible for the 2,4,6-TBA concentrations, while in places far away from the sea, long-range transport of 2,4,6-TBA occurred (Bidleman et al., 2023; Hsu et al., 2025; Zhan et al., 2023).

### Air concentrations of anthropogenic POPs (HCB, PCB 138, PCB 153) – compared to 2,4,6-TBA

Known sampling rates enabled converting HCB, PCB 138, and PCB 153 contents in the sampler into air concentrations (Eq. 1) (Armitage et al., 2013; Wania et al., 2003). Anthropogenic HCB (31–200 pg/m^3^), PCB 153 (24–66 pg/m^3^) and PCB 138 (16–43 pg/m^3^) were quantifiable in all six air samplers (Fig. 3b). A previous study on POPs in other Australian air samples from partly the same areas (Brisbane, Darwin, Phillip Island) but also twelve other regions was in the same range for HCB (32–170 pg/m^3^ (Li et al., 2023), recalculated using updated sampling rate), while PCB 153 contents were higher than previously reported in Australia (only up to 2 pg/m^3^) (Wang et al., 2015). However, the negligible PCB levels in the blank sample indicated no artifacts. Like with HNPs, POP contents were lowest in the remote inland sample from Idalia National Park (IDA) (Fig. 3b). Contrary to HNPs (but in line with the previous study (Wang et al., 2015)), the highest contents of these POPs were determined in urban Brisbane (BRI) (Fig. 3b). Interestingly, in the island samples, 2,4,6-TBA contents dominated over those of the POPs (Fig. 3b). Specifically, compared to HCB, 2,4,6-TBA contents were 1.0-, 1.5-, and even 3.5-fold higher in air from the island samples, with North Stradbroke Island (NSI) < Phillip Island (PHI) < One Tree Island (OTI) (Fig. 3b). Since 2,4,6-TBA and HCB’s vapor pressures (0.066 vs. 0.001 Pa) and Henry’s law constants (32–77 vs. 24–154 Pa*m^3^*mol^−1^) are in a similar range (Bidleman, 1984; Sander, 2023; Vetter et al., 2005), the distribution of both compounds in the environment should be comparable if the sources are close to each other. Hence, it is remarkable that, in air from North Stradbroke Island (NSI), which is located near Brisbane, 2,4,6-TBA was present in the same concentration as HCB, while in air from Brisbane (BRI), HCB was more than thrice as high as 2,4,6-TBA (Fig. 3b). This confirmed the expectations that the industrial chemical HCB was directly released in Brisbane (BRI) but sharply dropped within a short distance. Vice versa, this difference at the two sites further substantiated that 2,4,6-TBA was naturally produced and rather transported to Brisbane (BRI) and that its concentration in the Australian (coastal) environment was more prevalent than that of HCB.

### Further HNPs in the passive air samplers

Due to the lack of the corresponding sampling rates, further results on HNPs could only be stated in amounts accumulated in the samplers (Tab. S2). GC/ECNI-MS-SIM measurements indicated the presence of ~ ten polybrominated compounds in the early retention time range (Fig. 3a) of the sampler deployed at One Tree Island (OTI). Most of these compounds were also detected in the other samples, except for the inland sample (IDA). Unfortunately, structures could not be determined since their GC/ECNI-MS full scan spectra only featured low-mass fragment ions. Their highest abundance in the sample from One Tree Island (OTI) (31/42% of the intensity of 2,4,6-TBA, Fig. 3a) and a rather constant ratio to TBA in the other island samples (18%/4% in Phillip Island (PHI) and 29%/4% in North Stradbroke Island (NSI)) produced strong evidence that the two most abundant peaks from unknown compounds detected at ~ 12 min originated also from HNPs, with a hotspot of the natural producer in the GBR. The presence of two HNPs previously detected in air samples from other areas, i.e. 2,4-dibromoanisole (2,4-DBA) and 2,6-dibromoanisole (2,6-DBA) (Bidleman et al., 2017a, 2017b; Melcher et al., 2008; Pfeifer et al., 2001; Wittlinger & Ballschmiter, 1990; Wong et al., 2011), could not be confirmed by GC/ECNI-MS analysis using authentic reference standards.

The targeted analysis on 2,3,3',4,4',5,5'-heptachloro-1'-methyl-1,2'-bipyrrole (Q1, Fig. 2c) enabled its detection in samples collected from One Tree Island (OTI, GBR) and Phillips Island (PHI) but not in North Stradbroke Island (NSI) air (and also not in Brisbane and the other sites). Different from 2,4,6-TBA (2.0 vs. 6.2 ng/g adsorbent, factor ~ 0.3), the sample from Phillips Island (PHI) contained ~ five times more Q1 than the one from One Tree Island (OTI) (Tab. S3). Accordingly, Q1 reached 7% of 2,4,6-TBA at Phillips Island (PHI) and only ~ 0.5% at One Tree Island (OTI). However, the Henry’s law constant of Q1 (7.06 Pa*m^3^*mol^−1^) and 2,4,6-TBA (0.43 Pa*m^3^*mol^−1^) determined with the same method (Vetter et al., 2005) (note the different value for 2,4,6-TBA listed above) differed by a factor of ~ 16 (Vetter et al., 2005). Assuming a similar sampling rate as for PCB 153, Q1 concentrations would be in the low pg/m^3^ range. Distribution models indicated that the majority (~ 90%) of Q1 will be found in sediment and only ~ 3% in air (at 25 °C) (Vetter et al., 2005). High Q1 levels had previously been found in dolphins (450–9,100 ng/g lipids) and dugongs (5–160 ng/g lipids) from the same area as Brisbane (BRI), North Stradbroke Island (NSI), and One Tree Island (OTI) (Vetter et al., 2001). Given the high contents of Q1 in Australian marine biota samples, it is confirmed that only a small fraction of it is released into the air (One Tree Island, OTI), and this was the most plausible reason why Q1 was even undetectable on North Stradbroke Island (NSI).

Previously, Q1 was only reported once in air samples (here: active air samples from coastal Norway and the Antarctic) (Melcher et al., 2008; Vetter et al., 2000). However, in these active air samplers (with a high sampling volume of ~ 550–2000 m^3^), the Q1 amounts were in the fg/m^3^ range (< 1% of the 2,4,6-TBA content, (Melcher et al., 2008)). The extracted air volumes of the passive air samplers used here were only in the range of approximately 200 m^3^ leading to ~ 100 m^3^ per determination, depending on compound-specific sampling rates. This leads to the limit of detection being about an order of magnitude higher compared to the active air samplers collecting higher air volumes. Another possibility is that Q1 was mainly present in the particle-associated phase and not the gaseous phase in the atmosphere, leading to the detection by active samplers being favored compared to XAD passive samplers. Hence, it is likely that Q1 can only be detected by passive air samplers when levels in the marine environment are high, that is, in regions with a Q1 hotspot. Hitherto, such high Q1 levels were only found in samples from the Pacific Ocean, but research on HNPs such as Q1 is scarce. Our measurements confirm the prominent role of Q1 in the GBR and now, for the first time, in the Melbourne area due to the highest abundance of Q1 in the passive air sampler from this site.

In addition, 2′-methoxy-2,3′,4,5′-tetraBDE (2′-MeO-BDE 68 or BC-2, Fig. 1b) was only detected in air from One Tree Island (OTI, GBR) but not from Phillip Island (PHI) (Fig. 3a). The content (representing the mass accumulated in the passive sampler) of 2′-MeO-BDE 68 in the passive air sampler of One Tree Island (OTI) was ~ 25 times lower than that of 2,4,6-TBA and ~ eight times higher than that of Q1 (Fig. 3a). 2′-MeO-BDE 68 (and 6-methoxy-2,2′,4,4′-tetraBDE, 6-MeO-BDE 47 or BC-3) has already been determined in active air samples collected from South Korea and the Baltic Sea (Bidleman et al., 2017a; Kim et al., 2014). The levels of 2′-MeO-BDE 68 in the air above the Baltic Sea were 1000 times lower than those of 2,4,6-TBA (Bidleman et al., 2016, 2017a). Therefore, the total content of 2′-MeO-BDE 68 in the samplers was ~ 1–3 pg, while the total content of all MeO-BDEs in the active air samplers in the study from South Korea was 15–90 ng (Bidleman et al., 2016; Kim et al., 2014). Thus, this also showed that much lower quantities were present in these studies than those detected in the passive samplers. However, there is a sheer gradient since active samplers are collecting with high levels of enrichment, making the detection of the low concentrations in the air possible. Like for Q1, less air is sampled and therefore 2′-MeO-BDE 68 accumulated using passive air samplers, leading to it only being detected in those deployed along the coast near a hotspot in the same marine habitat. This again underlines the particular role of environments such as the GBR with relatively high biological activity and concentrations of the HNP 2′-MeO-BDE 68.

## Conclusions

Examination of the six passive air samples from Australia revealed HNPs in all investigated samples. Especially, the high concentrations of 2,4,6-TBA in the remote inland sample indicated that 2,4,6-TBA was transported over a longer distance in air from hotspots of its (indirect) natural production, such as in the GBR. In agreement with that, the 2,4,6-TBA levels were also high in the coastal cities (Brisbane and Darwin). Therefore, it is hypothesized that the natural product 2,4,6-TBA is ubiquitously present in Australian air. While PCBs should almost exclusively be released from urban sites, HNPs must be transported to them. It is therefore remarkable that 2,4,6-TBA was detected at similar levels as PCBs in the Australian cities. The 2,4,6-TBA content at One Tree Island (OTI) of 420 pg/m^3^ marked its highest air concentration reported so far. As addressed above, the warm climate at One Tree Island (OTI) could support this, since hot temperatures favor its distribution into the air. Amounts of Q1 and 2′-MeO-BDE 68 in only one or two of the passive air samplers from the island samples were attributed to their considerably lower vapor pressures. However, they may play a similarly prominent role as 2,4,6-TBA in the water phase. In this context, the region at Phillip Island (PHI) could represent a (hitherto unexplored) marine region with even higher contents of Q1, since the Q1/2,4,6-TBA ratio at this site was ~15 times higher than at One Tree Island (OTI) in the GBR.

## Supplementary Information

Below is the link to the electronic supplementary material.

ESM 1 (PDF 273 KB)

## Data Availability

Data is provided within the manuscript or supplementary information files.
