# Supplementary material for: The natural product 2,4,6-tribromoanisole is the predominant polyhalogenated compound in representative Australian passive air samples
Source: Environ Monit Assess. 2025 Oct 24;197(11):1242. doi: 10.1007/s10661-025-14638-7 (PMC12549748; doi:10.1007/s10661-025-14638-7)
Supplement: Supplementary file 1 — (PDF 273 KB) [file 10661_2025_14638_MOESM1_ESM.pdf]

Supplementary Information for

The natural product 2,4,6-tribromoanisole is the predominant polyhalogenated compound in representative Australian passive air samples

Sina Schweizer[1], Xianyu Wang[2], Chris Paxman[2], Jochen Müller[2] and Walter Vetter[1]*

[1] University of Hohenheim, Institute of Food Chemistry, Department of Food Chemistry (170b), 70599 Stuttgart, Germany

[2] University of Queensland, Queensland Alliance for Environmental Health Sciences, 4102 Brisbane, Australia

* Corresponding author

Walter Vetter

University of Hohenheim

Institute of Food Chemistry

Garbenstraße 28

D-70599 Stuttgart, Germany

E-mail: walter.vetter@uni-hohenheim.de

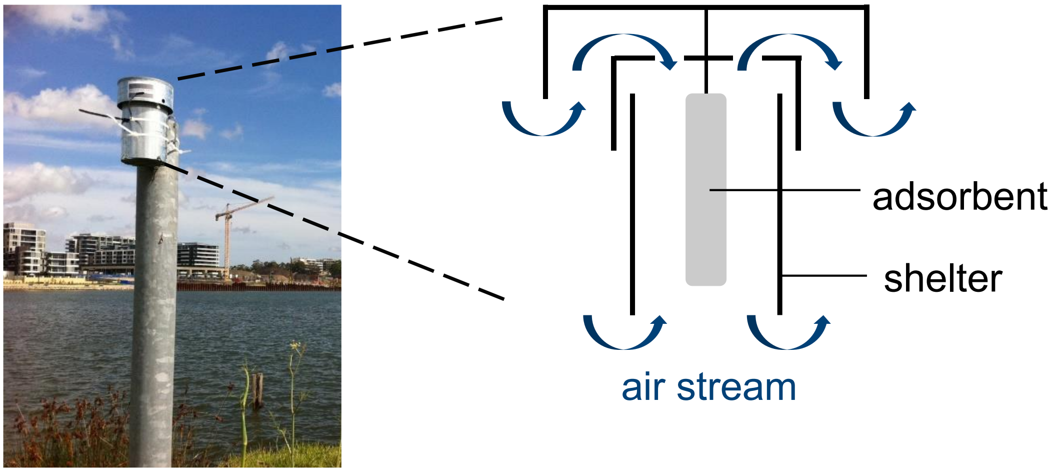

Fig. S1. Picture and schematic illustration of the used passive air samplers.

Tab. S1. Contents [ng/g adsorbent] of the HNPs in the air samplers from different regions of Australia.

CAS RN	Systematic chemical name	Abbreviation (used in this study)	Formula
607-99-8	2,4,6-tribromoanisole	2,4,5-TBA	C7H5Br3O
428442-17-5	2,3,3',4,4',5,5'-heptachloro-1'-methyl-1,2'-bipyrrole	Q1	C9H3Cl7N2
96920-28-4	2′-methoxy-2,3′,4,5′-tetraBDE	BC-2	C13H8Br4O2
118-74-1	hexachlorobenzene	HCB	C6Cl6
35065-28-2	2,2´,3,4,4´,5´-hexachlorobiphenyl	PCB 138	C12H4Cl6
35065-27-1	2,2´,4,4´,5,5´-hexachlorobiphenyl	PCB 153	C12H4Cl6

Tab. S2. Contents [ng/g adsorbent] of the HNPs in the air samplers from different regions of Australia.

Location	Year	Deployment [days]	Sample	Analyte	Content [ng/g adsorbent]	Total content sampler [ng]
Darwin[2] (Northern Territory)	2011	386	DAR_2_2 2011	2,4,6-TBA	1.37	13.7
North Stradbroke Island[1] (Queensland)	2020	368	NSI_20_1	2,4,6-TBA	1.36	13.6
One Tree Island[1] (Queensland)	2020	353	OTI_20_2	2,4,6-TBA	6.19	61.9
				Q1	0.03	0.3
				BC-2	0.25	2.5
Idalia National Park[3] (Queensland)	2020	325	IDA_20_3	2,4,6-TBA	0.23	2.3
Brisbane[2] (Queensland)	2020	367	BRI_20_4	2,4,6-TBA	0.93	9.3
Phillip Island[1] (Victoria)	2020	340	PHI_20_1	2,4,6-TBA	2.00	20
				Q1	0.14	1.4

[1] island (marine, remote)

[2] coastal city (urban)

[3] inland (remote)

Tab. S3. Content [pg/m^3] of 2,4,6-TBA determined in the air samples from different regions of Australia.

**Location**	**Sample**	**Content [pg/m^3]**
Darwin[2]	DAR_2_2 2011	85
North Stradbroke Island[1]	NSI_20_1	88
One Tree Island[1]	OTI_20_2	420
Idalia National Park[3]	IDA_20_3	17
Brisbane[2]	BRI_20_4	60
Phillip Island[1]	PHI_20_1	140

[1] island (marine, remote)

[2] coastal city (urban)

[3] inland (remote)

Tab. S4. Limit of detection (LOD) and limit of quantification (LOQ) of the halogenated natural products (HNPs) and anthropogenic persistent organic pollutants (POPs) determined by GC/ECNI-MS-SIM.

	**LOD [pg]**	**LOQ [pg]**
2,2'-diMeO-BB 80 (BC-1)	0.9	3.1
2'-MeO-BDE 68 (BC-2)	2.4	8.1
6-MeO-BDE 47 (BC-3)	1.5	5.2
2',6-diMeO-BDE 68 (BC-11)	2.0	6.8
2,4-DBP	1.6	5.3
2,6-DBP	5.9	20
2,4-DBA	2.8	9.5
2,4,6-TBP	3.0	9.9
2,4,6-TBA	0.3	0.9
Q1	0.07	0.2
HCB	0.01	0.04
α-HCH	0.02	0.05
β-HCH	0.5	1.6
γ-HCH	0.1	0.5
PCB 28	0.4	1.2
PCB 52	10	34
PCB 101	0.3	1.0
PCB 118	0.04	0.1
PCB 138	0.03	0.1
PCB 153	0.02	0.1
PCB 180	0.03	0.1
